# Overexpression of FoxM1 is associated with tumor progression in patients with clear cell renal cell carcinoma

**DOI:** 10.1186/1479-5876-10-200

**Published:** 2012-09-24

**Authors:** Yi-Jun Xue, Ri-Hai Xiao, Da-Zhi Long, Xiao-Feng Zou, Xiao-Ning Wang, Guo-Xi Zhang, Yuan-Hu Yuan, Geng-Qing Wu, Jun Yang, Yu-Ting Wu, Hui Xu, Fo-Lin Liu, Min Liu

**Affiliations:** 1 Department of Urology, First Affiliated Hospital of Gannan Medical University, No. 23, Qing Nian Road, Ganzhou, 341000, People’s Republic of China

**Keywords:** Renal cell carcinoma, FoxM1, Prognosis, Small interfering RNA

## Abstract

**Background:**

Fork head box M1 (FoxM1) is a proliferation-associated transcription factor essential for cell cycle progression. Numerous studies have documented that FoxM1 has multiple functions in tumorigenesis and its elevated levels are frequently associated with cancer progression. The present study was conducted to investigate the expression of FoxM1 and its prognostic significance in clear cell renal cell carcinoma (ccRCC). Meanwhile, the function of FoxM1 in human ccRCC was further investigated in cell culture models.

**Methods:**

Real-time quantitative PCR, western blot and immunohistochemistry were used to explore FoxM1 expression in ccRCC cell lines and primary ccRCC clinical specimens. FoxM1 expression was knocked down by small interfering RNA (siRNA) in Caki-1 and 786-O cells; proliferation, colony formation, cell cycle, migration, invasion, and angiogenesis were assayed.

**Results:**

FoxM1 expression was up-regulated in the majority of the ccRCC clinical tissue specimens at both mRNA and protein levels. Clinic pathological analysis showed that FoxM1 expression was significantly correlated with primary tumor stage (*P* <0.001), lymph node metastasis (*P* = 0.01), distant metastasis (*P* = 0.01), TNM stage (*P* < 0.001) and histological grade (*P* = 0.003). The Kaplan–Meier survival curves revealed that high FoxM1 expression was associated with poor prognosis in ccRCC patients (*P* < 0.001). FoxM1 expression was an independent prognostic marker of overall ccRCC patient survival in a multivariate analysis (*P* = 0.008). Experimentally, we found that down-regulation of FoxM1 inhibited cell proliferation and induced cell cycle arrest with reduced expression of cyclin B1, cyclin D1, and Cdk2, and increased expression of p21 and p27. Also, down-regulation of FoxM1 reduced expression and activity of matrix metalloproteinase-2 (MMP-2), MMP-9 and vascular endothelial growth factor (VEGF), resulting in the inhibition of migration, invasion, and angiogenesis.

**Conclusions:**

These results suggest that FoxM1 expression is likely to play important roles in ccRCC development and progression, and that FoxM1 is a prognostic biomarker and a promising therapeutic target for ccRCC.

## Background

Renal cell carcinoma (RCC) accounts for approximately 3% of all adult malignancies and represents the most lethal urological cancer [1]. Approximately 60,920 new cases of RCC were diagnosed in the United States in 2011, with an estimated 13,120 deaths [2]. Worldwide, the incidence of RCC is over 200,000 new cases annually, with over 100,000 deaths per year [3]. Clear cell RCC (ccRCC) is the most common histological subtype, comprising 70–80% of all RCC cases [4]. Nearly 25-30% of patients with RCC have evidence of metastases at initial presentation [5,6]. Although radical nephrectomy is effective to cure early and local RCCs, 30% of patients develop metastatic disease after surgery [7]. Patients with metastatic RCC face a dismal prognosis and have limited therapeutic options. Median survival in a recent cohort was only 1.5 years with fewer than 10% of patients surviving to 5 years [8]. Therefore, it is of paramount importance to better understand the pathogenesis of aggressive RCC in order to develop effective strategies for the prevention and treatment of RCC.

Fork head Box M1 (FoxM1) is a member of the Fork head Box family of transcription factors that share a conserved winged helix DNA binding domain [9]. FoxM1 is ubiquitously expressed in all proliferating cells, including many tumor-derived cell lines. In normal tissues, FoxM1 is detectable in progenitors with extensive proliferating capacity while its expression is extinguished in differentiated or resting cells [10,11]. FoxM1 is known to be a key cell cycle regulator of both the transition from G_1_ to S phase and the progression to mitosis by regulating transcription of cell cycle genes, including cyclin B1, cyclin D1, Cdc25A, Cdc25B, aurora B kinase, surviving, p21^Cip1^, and p27^Kip1^[12-17]. Loss of FoxM1 expression has been reported to generate mitotic spindle defects leading to mitotic catastrophe [16-18].

Recent data from several groups have highlighted that FoxM1 is up-regulated in a wide variety of cancers such as basal cell carcinomas, prostate cancer, glioblastomas, gastric cancer, breast cancer, and lung cancer [19-24]. More importantly, the increased expression of FoxM1 has been correlated with clinically aggressive behavior and patient survival in numerous human cancers [25-30]. Hence, FoxM1 not only promotes tumorigenesis by endowing proliferative capacity and leading to uncontrolled cell division at the early period of cancer development but also enhances other tumorigenic behaviors in other stages of cancer development. Indeed, recent evidence has implicated FoxM1 in several other cancer-related processes such as angiogenesis, invasion, and metastasis. For instance, FoxM1 was shown to stimulate invasion and angiogenesis of pancreatic cancer cells through induction of matrix metalloproteinase MMP-2 and MMP-9, as well as vascular endothelial growth factor (VEGF) [31]. Similar functions of FoxM1 in stimulating expression of MMP-2 and MMP-9 have also been documented in other malignancies, such as glioblastoma [32], breast carcinoma [33], and colorectal carcinoma [34]. Moreover, overexpression of FoxM1 coincides with metastasis of prostate cancer [35]. Furthermore, the mechanistic studies by Park et al. suggested that FoxM1 could function as a master activator of metastasis in nude mice, as it induced various steps of metastasis [36]. The study demonstrated that in the absence of Arf, FoxM1 overexpression contributes directly to metastatic behavior by driving the epithelial-mesenchymal transition through Akt, disrupting the rigidity of the cytoskeleton by upregulating the microtubule destabilizing protein Stathmin, and promoting the formation of pre-metastatic niches at distant organs by upregulating the lysyl oxidase collagen cross-linking proteins LOX and LOX2. These results indicate that FoxM1 may play diverse roles in cancer progression and that it could be a promising therapeutic target.

However, the expression pattern, clinical relevance, and biological function of FoxM1 in ccRCC have so far not been investigated. In the present study, we examined both mRNA and protein expression patterns in ccRCC tissues. We also investigated the correlations between FoxM1 expression and various clinic pathologic parameters, and its prognostic value for survival of patients with ccRCC. Then, we employed the small interfering RNA (siRNA) technique to evaluate the effects of knockdown of FoxM1 on proliferation, migration, invasion and angiogenesis of ccRCC cell lines *in vitro*. Together, our data highlight an important role for FoxM1 in controlling ccRCC progression.

## Methods

### Patients and surgical specimens

A total of 83 primary ccRCC tissues and matched adjacent nontumor renal tissues were obtained from patients who underwent radical nephrectomy in the Department of Urology, First Affiliated Hospital of Gannan Medical University between 2004 and 2008. None of the patients had received chemotherapy or radiotherapy before surgery. After surgical resection, tumor specimens and corresponding adjacent nontumor tissues were collected and stored in liquid nitrogen until use. Parts of each sample were fixed in formalin, embedded in paraffin and stored in the Department of Pathology, First Affiliated Hospital of Gannan Medical University. Fourty-five of these 83 patients were men and 38 were women. The median age of the patients was 57 years (range, 31-76 years). The median follow-up time was 53.2 months (range, 4-78 months). Information on gender, age, stage of disease, and histopathologic factors was abstracted from the medical records. All of the tumors were confirmed as ccRCC by the clinic pathologic department of the hospital. All of the cases were staged according to the tumor node metastasis staging system and nuclear grade was evaluated on the basis of the Fuhrman criteria. Patients’ data are summarized in Table 1. For the use of these clinical materials for research purposes, prior patient’s consent and approval from the Institute Research Ethics Committee were obtained.

**Table 1:** FoxM1 protein expression in 83 ccRCC tissues determined by immunohistochemistry

**Variable**	**Total**	**FoxM1 exression**		***P-*value**
		**Low**	**High**	
Age,years (median 57)				
<57	41	23	18	0.533
≥57	42	22	20	
Gender				
Male	51	27	24	0.473
Female	32	18	14	
T stage				
T_1-2_	61	41	20	<0.001
T_3-4_	22	4	18	
N stage				
N_0_	71	43	28	0.01
N_1-2_	12	2	10	
M stage				
M_0_	74	44	30	0.01
M_1_	9	1	8	
Histological grade				
G_1-2_	52	35	17	0.003
G_3-4_	31	10	21	
TNM stage				
I-II	56	59	17	<0.001
III-IV	27	6	21	

### Immunohistochemistry staining

All samples were fixed in 10% formaldehyde solution, embedded in paraffin blocks, cut in 4-μm-thick sections, and mounted on glass slides. Each slide was dewaxed in xylene and rehydrated in grade alcohol, followed by boiling in 10 mmol/L of citrate buffer (pH 6.0) for antigen retrieval. After inhibition of endogenous peroxidase activities for 30 minutes with methanol containing 0.3% H_2_O_2_, the sections were blocked with 2% bovine serum albumin for 30 minutes and incubated overnight at 4°C with primary polyclonal rabbit anti-human FoxM1 antibody (Santa Cruz Biotechnology Inc, Santa Cruz, CA, USA; 1: 50 dilution). After washing thrice with PBS, the slides were incubated with horseradish peroxidase-conjugated goat anti-rabbit IgG for 30 minutes, followed by reaction with diaminobenzidine and counterstaining with Mayer′ hematoxylin. Negative control was done by omission of the primary antibody and substituting it with nonspecific rabbit IgG.

### Evaluation of immunohistochemical staining

Three pathologists (H.Y., S.P. and X.H.) evaluated the immunostaining in a blinded fashion without any knowledge of the clinical outcome or other clinicopathological data. If there was a discrepancy in individual evaluations, then all the three pathologists reevaluated the slides together to reach a consensus. Immunohistochemical staining of FoxM1 was evaluated using a semi-quantitative scoring system for both staining intensity and the percentage of positive cells. A score was calculated by multiplying the intensity (negative scored as 0, mild scored as 1, moderate scored as 2 and strong scored as 3) by percentage of stained cells (0, < 5%; 1, 5–25%; 2, 26–50%; 3, 51–75%; and 4, 76–100%). Scores of multiplication were graded as follows: −, 0; +, 1–3; ++, 4–8; +++, 9–12. Additionally, for statistical analysis, the − and 1+ cases were pooled into the low-expression group, and the 2+ and 3+ cases were pooled into the high-expression group.

### Cell lines

Human RCC cell lines 786-O and Caki-1 were purchased from the American Type Culture Collection (Rockville, MD). Another three human RCC cell lines, A498, ACHN and OS-RC-2 were preserved in our institute. Immortalized normal human proximal tubule epithelial cell line HK-2 was obtained from the Cell Bank of Type Culture Collection of Chinese Academy of Sciences (Shanghai, China). HK-2 cells were cultured in K-SFM medium (Gibco Life Technologies, Grand Island, NY), and other cells were cultured in RPMI-1640 medium (HyClone Laboratories, Logan, UT) with 10% fetal bovine serum (Gibco Life Technologies, Grand Island, NY), 50U/ml of penicillin and 50 μg/ml of streptomycin. Human umbilical vein endothelial cells (HUVEC) were obtained from ScienCell Research Laboratories (Carlsbad, CA, USA) and cultured in ECM (Carlsbad, CA, USA). All cells were cultured in a sterile incubator maintained at 37°C with 5% CO_2_.

### Gene silencing using siRNA

FoxM1 siRNA (GGACCACUUUCCCUACUUU) and control siRNA (GGACCUGUAUGC GUACAUU) were purchased from Shanghai Genepharma Co. Ltd. (Shanghai, China). Cells were Transfected using Lipofectamine 2000 (Invitrogen, Carlsbad, CA, USA) according to the manufacturer’s instructions. Following transfection, the mRNA and protein levels were assessed 48 hours later.

### Real-time quantitative PCR

Total RNA was isolated from tissues and Transfected cells using TRIzol (Invitrogen) according to the manufacturer’s protocol. First-strand cDNAs were synthesized using the High Capacity cDNA Reverse Transcription Kit (Applied Bios stems, Foster City, CA, USA). Quantitative real-time PCR was performed using SYBR Green PCR Master Mix (Applied Bios stems) in a 7900 Real-Time PCR System (Applied Bios stems). β-actin was used as the reference gene. The following primers were used: for FoxM1, 5'-AACCGCTACTTGACATT GG-3' (forward), 5'-GCAGTGGCTTCATCTTCC-3' (reverse); for CyclinB1, 5'-GGTTGG GTCGGCCTCTACC T-3' (Forward), 5'-AGCCAGGTGCTGCATAACTGGAA-3' (Reverse); for CyclinD1, 5'-TCTACACCGACAACTCCATCCG-3' (Forward), 5'-TCTGGCATTTTGG AGAGGAAGTG-3' (Reverse); for CDK2, 5'-CTCCTGGGCTCGAAATATTATTCCACAG -3' (Forward), 5'-CCGGAAGAGCTGGTCAATCTCAGA-3' (Reverse); for p27, 5'-CGCT CGCCAGTCCATT-3' (Forward), 5'-ACAAAACCGAACAAAACAAAG-3' (Reverse); for p21, 5'-TCCAGCGACCTTCCTCATCCAC-3' (Forward), 5'-TCCATAGCCTCTACTGCCA CCATC-3' (Reverse); for MMP2, 5'-CCGTGGTGAGATCTTCTTCT-3' (Forward), 5'-CCTC GTATACCGCATCAATCT-3' (Reverse); for MMP9, 5'-TTCATCTTCCAAGGCCAATC-3' (Forward), 5'-CTTGCTGCTGCTAAAGTTCG-3' (Reverse); for VEGF, 5'-CTCTACCTCCA CCATGCCAAGT-3' (Forward), 5'-TGATTCTGCCCTCCTCCTTCT-3' (Reverse). The PCR cycles were 95°C for 10 minutes, followed by 40 cycles of 95°C for 15 seconds and 60°C for 1 minute. Each reaction was performed in triplicate and analyzed individually. The results were calculated by using 2^-∆∆Ct^ method.

### Western blot assay

Cells and tissues were lysed in lysis buffer containing protease inhibitor cocktail. Protein concentration was determined using a Bio-Rad protein assay system (Bio-Rad, Hercules, CA, USA). Equivalent amounts of proteins were separated by SDS-PAGE, and then transferred to polyvinylidene difluoride membranes (Bio-Rad). After being blocked in Tris buffered saline (TBS) containing 5% non-fatmilk, the membranes were incubated with specific primary antibodies (Santa Cruz Biotechnology, Santa Cruz, CA, USA) at 4°C for 12 hours and then with horseradish peroxidase-conjugated anti-rabbit antibody (Zhongshan, Beijing, China) for 2 hour at room temperature. Signals were detected on X-ray film using the ECL detection system (Pierce, Rockford, IL, USA). The relative protein levels were calculated based on β-actin as the loading control.

### MTT assay

Cells were plated in 96-well culture plates at about 5 × 10^3^ cells per well 24 hour after transfection. Then, 20 μl of 5 mg/ml MTT solution was added to each well and incubated for 4 hours at 37°C, the media was removed from each well, and the resultant MTT formazan was solubilized in 150 μl of DMSO. The absorbance values at 490 nm were measured using a microplate reader (Bio-Rad). The experiment was repeated three times and each experiment had six replicate wells.

### Colony formation assay

Cells were Transfected with control or FoxM1 siRNA for 48 hours and then plated at 1 × 10^3^ cells/well of a 6-well plate in triplicate. After 14 days of culture, the colonies were fixed with methanol and stained with crystal violet. The number of colonies per well was counted using a dissecting microscope with a threshold of 50 cells necessary to constitute a colony. At least two independent experiments were performed.

### Cell cycle analysis

Cells were harvested 48 hours after transfection with control or FoxM1 siRNA and fixed in 70% ice-cold ethanol overnight. The cells were then washed with PBS, and stained with propidium iodide (50 mg/ml) in PBS supplemented with RNase (50 mg/ml) in the dark at room temperature for 30 minutes. Tests were performed in triplicate for each sample, and analyses of cell cycle distribution were performed by flow cytometer (FACS CantoII, BD Bioscience, USA) in accordance with the manufacturer’s guidelines.

### Gelatin zymography

After transfection with control siRNA or FoxM1 siRNA for 24 hours, the complete medium was removed, and the cells were cultured in serum-free medium. After 24 hours, the conditioned medium was harvested, and then centrifuged to remove the cellular debris and separated by 8% acrylamide gels that contained 0.1% gelatin under non-reducing conditions. Gels were washed in 2.5% Triton X-100 and incubated overnight in 2.5% Triton X-100 solution at room temperature, with gentle agitation to remove SDS, and then were soaked in reaction buffer (50 mM Tris-HCl, 200 mM NaCl, 10 mM CaCl_2_) at 37°C overnight. After reaction, the gels were stained with 0.5% Coomassie Brilliant Blue solution, containing 20% methanol and 10% acetic acid, for 1 hour, distained with 20% methanol and 10% acetic acid, and visualized. The bands represent the results of gelatinase quantity and activity.

### Enzyme-linked immunosorbent assay (ELISA) for VEGF

Cells (1 × 10^5^) Transfected with control or FoxM1 siRNA were maintained in serum-free medium for 48 hours. The medium was collected, and the concentrations of VEGF in the medium were determined using an enzyme-linked immunosorbent assay (ELISA) kit (R&D systems, USA) according to the manufacturer' instruction.

### Scratch migration assay

Cells were seeded to 12-well plates and Transfected with control or FoxM1 siRNA. At 24 hours after transfection, cells were scratched using the tip of a sterile 200-μl pipette (width: ~1 mm) in each well. The plates were washed twice with PBS in order to remove the detached cells, and incubated at 37°C in 5% CO_2_. Wound closure was monitored at various time points by observation under a microscope and the degree of cell migration was quantified by the ratio of gap distance at 24 hours to that at 0 hour. The experiment was done in triplicate.

### Matrigel invasion assay

Cell invasion assay was performed using a 24-well Tran swell chamber with a pore size of 8 μm (Costar, New York, NY, USA). The inserts were coated with 50 μl Matrigel (dilution at 1: 2; BD Bioscience, Franklin Lakes, NJ, USA). Cells were trypsinised after transfection with control or FoxM1 siRNA for 48 hours and transferred to the upper Matrigel chamber in 100 μl of serum free medium containing 1 × 10^5^ cells and incubated for 24 hours. The lower chamber was filled with medium that contained 10% fetal bovine serum as chemoattractants. After incubation, the noninvaded cells on the upper membrane surface were removed with a cotton tip, and the cells that passed through the filter were fixed and stained using 0.1% crystal violet. The numbers of invaded cells were counted in five randomly selected high power fields under a microscope. This experiment was performed in triplicate.

### Matrigel in vitro HUVEC tube formation assay

Cells Transfected with control or FoxM1 siRNA were cultured in serum-free RPMI 1640 for 24 hours. The conditioned medium were collected, centrifuged and stored at -20°C until assay. HUVEC (1 × 10^5^ cells/well) in 500 μl of the indicated conditioned medium were seeded onto a 24-well plate, which was precoated with 100 μl of growth factor-reduced Matrigel (BD Bioscience) for 30 minutes. Following stimulation with the cell conditioned medium for 12 hours, tube formation was observed under an inverted microscope and counted. The number of tube formations was measured by counting the number of tube-like structures formed by connected endothelial cells in five randomly selected fields under a microscope. The assay was performed in triplicate.

### Statistical analysis

The statistical analyses were performed using the Statistical Package for the Social Sciences, version 16.0 (SPSS Inc., Chicago, IL, USA). A paired-samples t-test was used to compare FoxM1 mRNA and protein expression in the ccRCC tissues with that of their paired adjacent nontumor tissue samples. The relationship between FoxM1 protein expression and the clinicopathological features was analyzed using χ2 tests. Overall survival curves were calculated with the Kaplan-Meier method and were analyzed with the log-rank test. A Cox proportionalhazards analysis was used in univariate and multivariate analyses to explore the effects of FoxM1 expression and ccRCC clinicopathological variables on survival. Unpaired 2-tailed Student's t-tests were used to analyze comparisons between the 2 groups. A *P*-value of < 0.05 was regarded as statistically significant.

## Results

### FoxM1 mRNA and protein expression in primary ccRCC tissue samples and RCC cell lines

**Figure 1:**
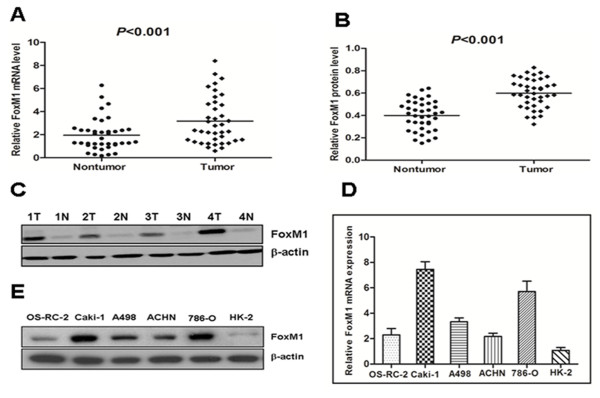
**The expression of FoxM1 mRNA and protein in the human ccRCC surgical specimens and RCC cell lines, as evaluated by real-time quantitative PCR and western blot.A**, The relative mRNA expression of FoxM1 was higher in 39 ccRCC tumor tissues than in matched adjacent nontumorous tissues (P < 0.001). **B***,* The FoxM1 protein expression was higher in the tumor tissues than in matched adjacent nontumorous tissues (P < 0.001). **C**, Expression of FoxM1 protein in four representative pairs of ccRCC tissues is presented. N, nontumorous tissues; T, ccRCC tissues. **D**, The FoxM1 mRNA expression in human RCC cell lines was higher in the OS-RC-2, Caki-1, A498, ACHN and 786-O cells, particularly in the Caki-1 and 786-O cells, compared with the normal proximal tubule epithelial cell line HK-2. **E**, The FoxM1 protein expression was elevated in the OS-RC-2, Caki-1, A498, ACHN and 786-O cells compared to the normal proximal tubule epithelial cell line HK-2.

We first examined FoxM1 mRNA expression in 39 paired clinical samples from ccRCC patients (tumor tissues and matched adjacent nontumor tissues) by real-time quantitative PCR. The results revealed a statistically significant elevation of FoxM1 mRNA in tumors, as compared to the matched adjacent nontumor tissues (*P* < 0.001, Figure 1A). To investigate whether FoxM1 was also elevated at the protein level, western blot was performed on those 39 paired ccRCC clinical samples. We found that the protein level of FoxM1 in tumor tissues was significantly higher than that in adjacent nontumor tissues (*P* < 0.001, Figure 1B), consistent with the results of real-time quantitative PCR. The protein level of FoxM1 in four representative pairs of samples is shown in Figure 1C. We also used real-time quantitative PCR and western blot to detect the expression of FoxM1 mRNA and protein in RCC cell lines as well as in an immortalized normal human proximal tubule epithelial cell line. As shown in Figure 1D, the OS-RC-2, Caki-1, A498, ACHN and 786-O showed higher FoxM1 transcript levels relative to the HK-2 normal proximal tubule epithelial cell line (Figure 1D). Likewise, FoxM1 protein expression was elevated in those RCC cell lines compared to the HK-2 cell line (Figure 1E).

### Immunohistochemical analysis of FoxM1 expression in ccRCC clinical samples and its relationship to clinicopathological parameters

**Figure 2:**
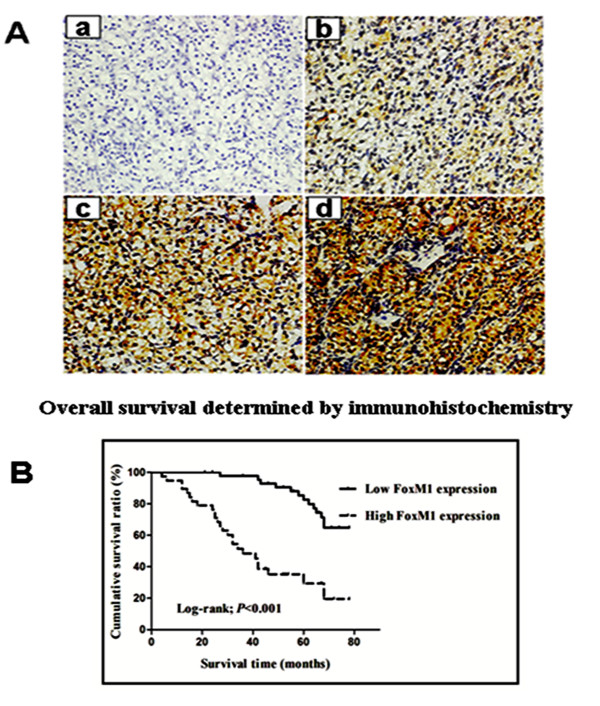
**FoxM1 protein expression and patient survival.A**, Immunohistochemical analysis of FoxM1 protein expression in 83 cases of ccRCC tissues: – in a, 1+ in b, 2+ in c, and 3+ in d. Magnification, all × 200. **B***,* Overall survival analysis using the Kaplan–Meier method revealed that patients with high FoxM1 expression had obviously lower overall survival rates than did those with low FoxM1 expression.

We further analyzed FoxM1 protein level in 83 ccRCC tissues and adjacent nontumor tissues using an immunohistochemical approach. FoxM1 protein expression in tumors was usually increased compared with that in adjacent nontumor tissues. FoxM1 stained mainly in the cytoplasm of the cells (Figure 2A b-d). 45 (54.2%) cases showed low FoxM1 expression (FoxM1− or FoxM1+), and 38 (45.8%) cases exhibited high FoxM1 expression (FoxM1++ or FoxM1+++). The relationship between FoxM1 expression and various clinicopathological parameters is described in Table 1. FoxM1 staining level significantly correlated with primary tumor stage (*P* < 0.001), lymph node metastasis (*P* = 0.01), distant metastasis (*P* = 0.01), TNM stage (*P* < 0.001) and histological grade (*P* = 0.003). There was no significant association between FoxM1 expression and patients’ gender and age.

### FoxM1 expression and patient survival

The prognostic value of FoxM1 for overall survival in ccRCC patients was evaluated by comparing the patients with high and low FoxM1 expression. According to the Kaplan–Meier survival analysis, ccRCC patients with high FoxM1 expression had obviously lower overall survival rates than did those with low FoxM1 expression (Figure 2B, Log-rank value =27.484, *P* < 0.001). Univariate and multivariate analyses were conducted using Cox proportional hazards model to examine the impact of FoxM1 expression and other clinicopathological parameters in ccRCC patients. FoxM1 expression (*P* < 0.001), primary tumor stage (*P* < 0.001), lymph nodes metastasis (*P* = 0.007), distant metastasis (*P* < 0.001) and histological grade (*P* = 0.01) were significant prognostic factors in the univariate analysis (Table 2). Multivariate Cox regression analyses showed that advanced primary tumor stage (*P* = 0.001), distant metastasis (*P* = 0.025) and high FoxM1 expression (*P* = 0.008) were independent prognostic factors (Table 2). Thus, FoxM1 expression may be useful for predicting the overall survival of ccRCC patients.

**Table 2:** Prognostic factors in Cox proportional hazards model

**Variable**	**Univariate analysis**			**Multivariate analysis**		
	**Risk ratio**	**95% CI**	***P***	**Risk ratio**	**95% CI**	***P***
Age	0.958	0.506-1.811	0.894			
≥57 vs <57						
Gender	0.978	0.468-2.042	0.953			
male vs female						
Histological grade	2.317	1.224-4.388	0.01			
G_3-4_ vs G_1-2_						
Lymph node status	2.787	3.317-5.899	0.007			
N_1-2_ vs N_0_						
Primary tumour stage	6.295	3.190-12.421	<0.001	4.336	1.859-10.116	0.001
T_3-4_ vs T_1-2_						
Distant metastasis	8.951	3.828-20.930	<0.001	2.950	1.149-7.573	0.025
M_1_ vs M_0_						
FoxM1	5.505	2.728-11.109	<0.001	3.034	1.337-6.887	0.008
high vs low						

### Effects of FoxM1 depletion on cell growth

**Figure 3:**
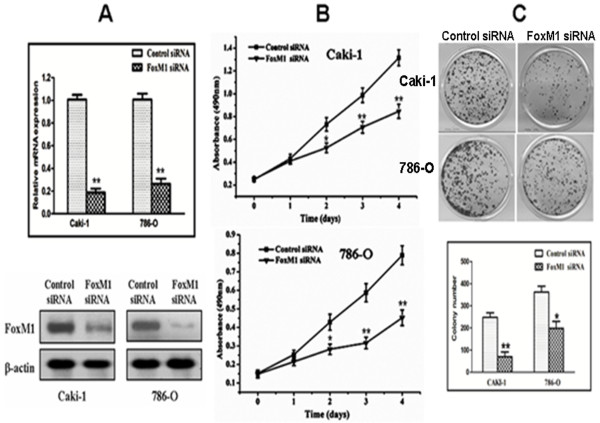
**Effects of FoxM1 depletion on cell growth.A,** FoxM1 mRNA levels were down-regulated by FoxM1 siRNA. **B***,* FoxM1 protein levels were down-regulated by siRNA. **C***,* Inhibition of cancer cell proliferation by FoxM1 siRNA tested by MTT assay. **D***,* Inhibition of cancer cell colony formation capacity by FoxM1 siRNA. Experiments were repeated at least three times, and representative data are presented; *bars*, SD.*, *P* < 0.05; **, *P* < 0.01, relative to control.

In order to determine whether FoxM1 could be an effective therapeutic target for ccRCC, we employed an RNA interference approach to knock down its expression in Caki-1 and 786-O cells expressing high levels of endogenous FoxM1. The efficacy of FoxM1 siRNA for knockdown of FoxM1 mRNA and protein was confirmed by real-time PCR and western blot analysis, respectively. We observed that FoxM1 mRNA levels were significantly reduced in cells Transfected with specific siRNA for FoxM1 compared with those Transfected with control siRNA (*P* < 0.01; Figure 3A). Also, the expression of FoxM1 protein was significantly decreased compared with the control siRNA-Transfected cells. Thus, the FoxM1 siRNA could effectively knock down FoxM1 expression at both transcriptional and translational levels. We next studied the impact of FoxM1 silencing on cell proliferation. The results of the MTT assay showed that down-regulation of FoxM1 significantly reduced the proliferation rate in both the cell lines tested compared with the control siRNA-Transfected cells (*P* < 0.01; Figure 3B). Colony formation assay further showed that down-regulation of FoxM1 in two tested cell lines with transfection of FoxM1 siRNA resulted in a clear reduction of the colony formation capacity compared with the control siRNA-Transfected cells (*P* < 0.05; Figure 3C). These results from colony formation assay are consistent with the MTT data, suggesting that FoxM1 expression influences the growth and proliferation of ccRCC cells.

### Effect of FoxM1 deletion on cell cycle

**Figure 4:**
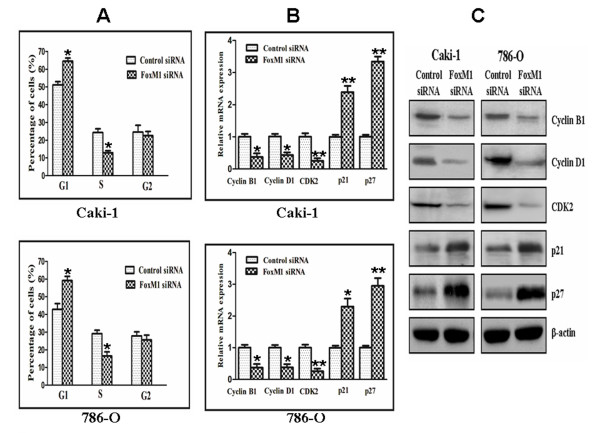
**Effect of FoxM1 deletion on cell cycle.A,** The cell cycle distribution was analyzed using propidium iodide staining and flow cytometry. *B and C,* The expression level of several known cell cycle regulatory factors as detected by real-time quantitative PCR (**B**) and Western blotting (**C**), respectively. The experiments were repeated thrice. *, *P* < 0.05; **, *P* < 0.01, relative to control.

Cell cycle analysis revealed that FoxM1 silencing in Caki-1 and 786-O cells caused a accumulation of cells in the G_0_-G_1_ phase and a decrease in the S phase compared with control siRNA-Transfected cells (*P* < 0.05; Figure 4A). To investigate the mechanism underlying the cell cycle arrest, we examined the levels of a few cell cycle regulatory factors and studied the effects of down-regulation of FoxM1. As shown in Figure 4B and 4C, the expression of cyclin B1, cyclin D1, and cyclin-dependent kinase 2 (Cdk2) at both the mRNA and protein levels was found to be decreased in cells Transfected with FoxM1 siRNA compared with those Transfected with control siRNA (*P* < 0.05). In contrast, down-regulation of FoxM1 was found to result in an increase in the expression of cyclin-dependent kinase inhibitors such as p21 and p27. Taken together, these results indicated that down-regulation of FoxM1 expression suppressed cell cycle progression in ccRCC cells.

### Effect of FoxM1 deletion on MMP-2, MMP-9 and VEGF

**Figure 5:**
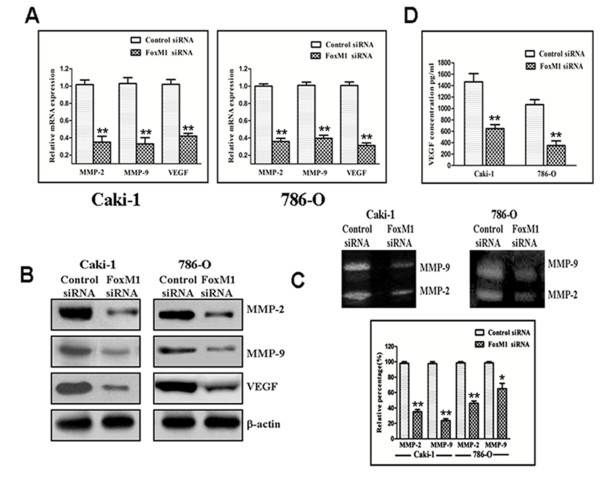
**Effect of FoxM1 deletion on the expression of various cell cycle regulatory factors and MMP-2, MMP-9, and VEGF.A** and **B**, real-time quantitative PCR and Western blot analysis showed that FoxM1 siRNA inhibited the expression of MMP-2, MMP-9, and VEGF at mRNA and protein levels in Caki-1 and 786-O cells. **C**, FoxM1 siRNA inhibited the activity of MMP-2 and MMP-9 based on gelatin zymography assay in Caki-1 and 786-O cells. **D**, FoxM1 siRNA inhibited the activity of VEGF in Caki-1 and 786-O cells. The experiments were repeated thrice. *, *P* < 0.05; **, *P* < 0.01, relative to control.

As shown in Figure 5A, real-time quantitative PCR analysis demonstrated that FoxM1 knockdown significantly decreased MMP-2, MMP-9 and VEGF mRNA expression compared with control siRNA-Transfected cells (*P* < 0.01). Similar results were observed by western blot analysis as well (Figure 5B). Next, we examined whether the down-regulation of FoxM1 could also lead to a decrease in MMP-2, MMP-9 and VEGF activity. As shown in Figure 5C, both MMP-2 (Caki-1and 786-O*, P* < 0.01) and MMP-9 (*P* < 0.01 for Caki-1 and *P* < 0.05 for 786-O) activities were decreased in the FoxM1 siRNA-Transfected cells, as determined by gelatin zymography when compared with control siRNA-Transfected cells. We also found that VEGF activity was significantly reduced by the down-regulation of FoxM1, as measured by ELISA when compared with control siRNA-Transfected cells (Figure 5D; *P* < 0.01). These results clearly suggest that tumor progression could be attenuated by the down-regulation of FoxM1.

### Effect of FoxM1 deletion on migration and invasion

**Figure 6:**
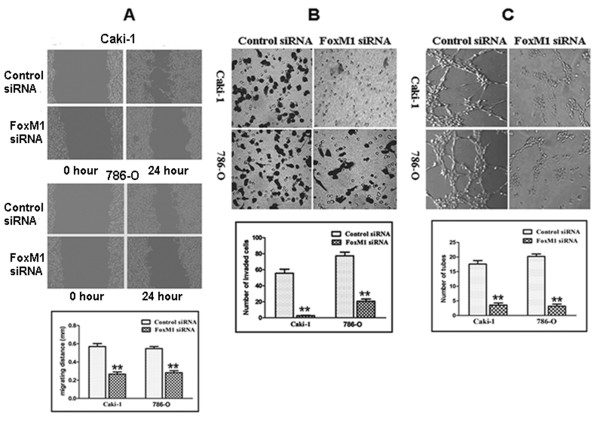
**FoxM1 siRNA decreased migration and invasion of Caki-1 and 786-O cells, and reduced the HUVECs tube formation.A***,* Scratch migration assay showing that FoxM1 siRNA decreased cell migration. **B***,* Matrigel invasion assay showing that FoxM1 siRNA-transfected cells resulted in low penetration through the Matrigel-coated membrane, compared with control cells. **C***,* Conditioned media from FoxM1 siRNA-transfected cells were able to significantly reduce the tube formation of HUVECs compared with the medium from control cells. The experiments were repeated thrice. **, *P* < 0.01, relative to control.

Because FoxM1 silencing inhibited the expression and activity of MMP-2, MMP-9 and VEGF that are thought to be critically involved in the processes of tumor cell migration, invasion and metastasis, we tested the effect of FoxM1 deletion on cancer cell migration and invasion. In the scratch migration assay, down-regulation of FoxM1 significantly suppressed the migration of both Caki-1 and 786-O cells (Figure 6A). The migrating distance of Caki-1 cells was 0.571 ± 0.055 mm in the control siRNA group and 0.267 ± 0.041 mm in the FoxM1 siRNA group (*P* < 0.01). In the 786-O cells, the migrating distance was 0.547 ± 0.040 mm in the control siRNA group and 0.283 ± 0.035 mm in the FoxM1 siRNA group (*P* < 0.01). Matrigel invasion assay showed that down-regulation of FoxM1 significantly suppressed the invasiveness of both cancer cells (Figure 6B). The average cell counts crossing matrigel-coated membrane in one high power field was 55.7. ± 8.7 for the control siRNA group and 2.3 ± 0.6 for the FoxM1 siRNA group of Caki-1 cells (*P* < 0.01); 77.3 ± 8.1 for the control siRNA group and 20.6 ± 4.5 for the FoxM1 siRNA group of 786-O cells (*P* < 0.01).

### Effect of FoxM1 deletion on angiogenesis

Because FoxM1 siRNA inhibited VEGF expression and activity, we tested whether FoxM1 siRNA-Transfected cells could reduce the tube formation of HUVECs cultured with conditioned medium (CM), an indirect measure of angiogenesis. As illustrated in Figure 6C, the CM obtained from the FoxM1 siRNA-Transfected cells showed significantly decreased tube formation per microscopic field as compared to control siRNA-Transfected cells (Caki-1 control *vs* FoxM1 siRNA: 17.6 ± 2.7 *vs* 3.6 ± 1.5, *P* < 0.01; 786-O control *vs* FoxM1 siRNA: 20.2 ± 1.9 *vs* 3.2 ± 1.6, *P* < 0.01).

## Discussion

Convincing evidence has shown that FoxM1 is upregulated in a wide variety of malignant tumors. FoxM1 overexpression has also been reported to be associated with worse prognosis and to serve as a prognostic marker in numerous types of human cancers. However, little is known about its expression pattern and biological significance in ccRCC. In the current study, we showed that FoxM1 expression determined by real-time quantitative PCR and Western blot was significantly higher in ccRCC tissues than that in adjacent nontumor renal tissues. Immunohistochemical analysis also confirmed that tumor tissues exhibited abundant FoxM1 expression, in contrast to adjacent nontumor tissues which displayed absence or lower FoxM1 expression. To investigate whether FoxM1 expression might be associated with the progression of ccRCC, the FoxM1 expression levels and the clinic pathologic characteristics of 83 patients with ccRCC were compared by immunohistochemistry. We found that high FoxM1 expression is significantly correlated with primary tumor stage, lymph node metastasis, distant metastasis, TNM stage, and histological grade, suggesting that its expression might be important for the acquirement of malignant potential in ccRCCs. Furthermore, elevated FoxM1 expression was identified as an independent worse prognostic factor in ccRCC patients. These findings are in agreement with studies in other human cancers overexpressing FoxM1 [25-30].

We have clearly shown that FoxM1 is highly expressed in ccRCC cells from patient samples. This prompted us to examine the biological function of FoxM1 in greater detail through in vitro analysis of ccRCC cell lines. Therefore, we first checked its expression level in several cell lines and picked up Caki-1 and 786-O with relatively high FoxM1 level for further study. We employed siRNA to knockdown FoxM1 expression in these two cell lines. We found an impaired proliferation capacity and colony formation ability of both Caki-1 and 786-O cells after FoxM1 knockdown. We also found that down-regulation of FoxM1 could inhibit cell migration, invasion, and angiogenesis. Thus, our study suggested that FoxM1 is a potential therapeutic target for the treatment of ccRCC.

Abnormal cell proliferation and growth are characteristics of cancer, including ccRCC. Most of the proliferative factors influence cell growth by affecting cell cycle progression. The importance of FoxM1 with respect to the cell cycle is well recognized. In the present study, cell cycle analyses revealed that FoxM1 knockdown cells showed higher levels of G_1_ phase and lower S phase than the control cells. So FoxM1 knockdown inhibited G_1_ to S transition in cell cycle progression, which might explain the mechanism of FoxM1 on ccRCC cell proliferation. Furthermore, we found that down-regulation of FoxM1 caused a marked reduction in cyclin B1, cyclin D1, and Cdk2 expression, which play important roles in cell cycle progression. We also observed an increased expression of cyclin-dependent kinase inhibitors such as p21 and p27 in FoxM1 siRNA-Transfected cells, which are known to negatively regulate cell cycle progression. These results suggest that FoxM1 influences the cell cycle progression by positively regulating the factors that favor cell cycle progression and also by negatively influencing the inhibitors of cell cycle in ccRCC cells.

Metastasis is an important aspect of ccRCC. It is known that MMPs are involved crucially in the processes of tumor cell invasion and metastasis [37,38]. Among these MMPs, MMP-2 and MMP-9 are directly linked with angiogenesis and degradation of the basement membrane collagen, and their expression and activity are correlated with metastatic abilities and prognosis of cancer [39,40]. FoxM1 has been shown to be associated with MMP-2 and MMP-9 in multiple tumor types [31-34]. Here, we showed that down-regulation of FoxM1 by siRNA in Caki-1 and 786-O cells led to reduced expression of MMP-2 and MMP-9. We also found that down-regulation of FoxM1 decreased MMP-2 and MMP-9 activity in the culture medium based on gelatin zymography assay. These results suggest that the suppression of FoxM1 expression has potential for antimetastatic therapy, at least in part, by inhibiting expression/activity of MMPs.

VEGF is another important factor in tumor cell invasion, angiogenesis, and metastasis. It is well documented that VEGF is a key mediator of angiogenesis and regulates most of the steps in the angiogenic signal cascade [41]. Several recent reports have documented a positive correlation between expression of FoxM1 and VEGF [31,33,34]. In the present study, we found a significant reduction in VEGF expression and activity by down-regulation of FoxM1 using siRNA transfection. These data suggest that the suppression of FoxM1 expression has potential for antimetastatic therapy, at least in part, by inhibiting expression/activity of VEGF.

In order to fully understand the consequences of such down-regulation in the expression and the activity of MMP-2, MMP-9 and VEGF, we performed scratch migration assay and matrigel invasion assay of ccRCC cells and tube formation assay of HUVECs. We found that down-regulation of FoxM1 led to a significant reduction in the migration and invasive potential of Caki-1 and 786-O cells and the tube formation of HUVECs. These results are consistent with the inactivation of MMP-2, MMP-9, and VEGF by the down-regulation of FoxM1, which inhibits cancer cell migration, invasion and angiogenesis. We recognize some limitations in the article. First, the precise molecular mechanisms of metastasis promotion by FoxM1 in ccRCC need to be further elucidated. Second, the *in vivo* metastasis assay should be performed to further testify the roles of FoxM1 in metastasis of human ccRCC.

## Conclusions

In summary, the present study firstly showed that FoxM1 expression was up-regulated in the majority of the ccRCC clinical tissue specimens at both mRNA and protein levels. Higher expression of FoxM1 positively correlates with the aggressive phenotype of ccRCCs, and predicts poor survival outcome of patients. We have also presented experimental evidence that down-regulation of FoxM1 in ccRCC cell lines using siRNA inhibited cell proliferation and induced cell cycle arrest with reduced expression of cyclin B1, cyclin D1, and Cdk2, and increased expression of p21 and p27. Furthermore, down-regulation of FoxM1 reduced expression and activity of MMP-2, MMP-9, and VEGF, resulting in the inhibition of migration, invasion, and angiogenesis. Based on these findings, we conclude that FoxM1 is functionally important in the development and progression of ccRCC and may serve as a new target for ccRCC therapy.

## Abbreviations

ccRCC: Clear cell renal cell carcinoma; FoxM1: Fork head box M1; MMP-2: Matrix metalloproteinase-2; MMP-9: Matrix metalloproteinase-9; VEGF: Vascular endothelial growth factor; ELISA: Enzyme-linked immunosorbent assay.

## Misc

Yi-Jun Xue, Ri-Hai Xiao and Da-Zhi Long contributed equally to this work

## Competing interests

The authors declare that they have no competing interests.

## Authors’ contributions

YJX and XFZ are responsible for the study design. YJX, RHX, and XYW performed the experiments and draft the manuscript. GXZ, YHY and GQW collected the data. DZL, JY, YTW, HX, FLL, and ML participated in the data analysis and interpretation. All authors read and approved the final manuscript.
